# Food insecurity, acculturation and diagnosis of CHD and related health outcomes among immigrant adults in the USA

**DOI:** 10.1017/S1368980019001952

**Published:** 2019-08-13

**Authors:** Michael D Smith, Alisha Coleman-Jensen

**Affiliations:** Economic Research Service, United States Department of Agriculture, 355 E. Street SW, Washington, DC 20024, USA

**Keywords:** Food security, Acculturation, Healthy immigrant effect, Cardiometabolic health, Heart-related health

## Abstract

**Objective:**

To deepen understanding of the relationship between food insecurity, acculturation, and diagnosis of CHD and related health outcomes among immigrant adults.

**Design:**

Using cross-sectional, nationally representative data from the National Health Interview Survey 2011 to 2015, we address two research questions. First, what is the relationship of household food insecurity and acculturation with: CHD, angina pectoris, heart attack, self-rated poor health and obesity? Second, what is the association of food insecurity with these health outcomes over years of living in the USA? We estimate multivariate logistic regressions without (question 1) and with (question 2) an interaction term between food insecurity and acculturation for CHD and related health outcomes.

**Setting:**

USA.

**Participants:**

Low-income immigrant adults.

**Results:**

Food insecurity and acculturation are both associated with diagnosis of CHD and related health outcomes among immigrant adults. Food insecurity and acculturation are associated with the health of female immigrants more than males. Also, the differences by food security status in the probability of having several poor health outcomes (self-rated heath, obesity, women’s angina pectoris) are largest for those in the USA for less than 5 years, decrease for those who have lived in the USA for 5–14 years, and are larger again for those in the USA for 15 or more years.

**Conclusions:**

Recent and long-term food-insecure immigrants are more vulnerable to CHD and related health outcomes than those in the USA for 5–14 years. Further research is needed to understand why.

The immigrant population in the USA has increased significantly over the last several decades. Immigrants were 14 % of the US population in 2013, up from 8 % in 1990^(^ ^1^ ^)^. Research on the determinants of immigrant health shows that US immigrants tend to have a combination of good health and low socio-economic status upon their arrival, but this health advantage over their socio-economic native-born peers tends to decline the longer they live in the USA.† Acculturation, then, measured by the number of years living in the USA,‡ is associated with poorer health. Exposure to the US environment often leads immigrants to adopt American lifestyles and dietary behaviours^(^ ^6^ ^)^. These dietary and behavioural changes can have important health implications for immigrants over time, especially for the food insecure.

Households are considered food secure if they have access at all times to the foods necessary for all members to enjoy an active, healthy life^(^ ^7^ ^)^. Food insecurity entails having difficulty consistently obtaining adequate food because of limited economic resources. Many factors make immigrants more likely to experience food insecurity than the native-born population^(^ ^8^ ^–^ ^13^ ^)^. For example, their immigrant status may affect their access to federal assistance programmes^(^ ^8^ ^,^ ^14^ ^–^ ^16^ ^)^. Low-wage employment, limited education (i.e. lack of a high-school diploma), job insecurity, language barriers and marginal social standing can be common among immigrants^(^ ^11^ ^)^. These economic constraints can lead to uncertain access to food and may also contribute to health inequalities^(^ ^17^ ^–^ ^19^ ^)^.

Food insecurity is associated with a range of negative health outcomes among native-born US adults. These include chronic diseases such as stroke, cancer and asthma^(^ ^19^ ^–^ ^21^ ^)^ and cardiometabolic conditions such as CHD, diabetes, obesity and hypertension^(^ ^22^ ^–^ ^29^ ^)^. Food insecurity also makes managing these conditions difficult^(^ ^22^ ^)^. For example, due to the higher cost of whole grains, lean proteins and fresh produce, which are a critical part of preventing and managing such conditions, they are less often consumed in the diets of the food insecure^(^ ^22^ ^,^ ^24^ ^,^ ^30^ ^)^.

Most research on food security and heart-related health has not distinguished immigrants from the native born, whether it is directed at adults^(^ ^20^ ^)^ or children^(^ ^31^ ^)^. Yet their relative advantage over their socio-economic peers, in terms of health condition upon arrival, and their deterioration over years of living in the USA, suggest that food insecurity may affect the health of immigrants differently from the population overall. Using repeated cross-sectional data from the National Health Interview Survey (NHIS) from 2011 to 2015, we address two research questions. First, what is the relationship of household food insecurity and acculturation with diagnosis of CHD and related health outcomes, namely angina pectoris, heart attack, self-rated poor health and obesity? These health conditions are relevant to public health policy given their preventability and their prevalence, cost and morbidity in the USA^(^ ^5^ ^,^ ^20^ ^,^ ^32^ ^)^. Second, what is the association of food insecurity with CHD and related outcomes over years of living in the USA?

To answer these questions, we first estimate a series of multivariate logistic regression models examining the associations of household food insecurity and acculturation separately with each of the five health outcomes. Second, we estimate the logistic regressions with an interaction term between food insecurity and acculturation. We also examine heterogeneity across the severity of food insecurity to see if the relationships between household food insecurity and CHD and related outcomes change with the severity of food insecurity. Identifying the relationships of food insecurity and acculturation with CHD and related health outcomes among immigrant adults is an important step towards the development of appropriate health intervention strategies.

## Data

The analyses in the present paper use pooled cross-sectional data from the 2011 to 2015 waves of the NHIS, an in-person health survey of the civilian non-institutionalized population of the USA conducted by the US Census Bureau for the National Center for Health Statistics. The sampling strategy follows a multistage area probability design that, when weighted, results in a nationally representative sample. The NHIS collects detailed information on demographics and topics including health status and limitations, injuries, health-care access and utilization, health insurance coverage, immigration status, and income and assets. Beginning in 2011, the US Department of Agriculture sponsored the inclusion of the ten-item, adult 30 d food security module in the NHIS.

The sample consists of working-age immigrant adults aged 18 to 64 years in households at or below 300 % of the federal poverty line. Following Gregory and Coleman-Jensen^(^ ^20^ ^)^, we exclude retirement-age respondents because of their differences in time allocation, income and health insurance from working-age adults. We also exclude higher-income households to compare food-insecure households with other low-income non-food-insecure households with similar resource constraints. Our interest is the heart-related health outcomes of immigrants and not the comparison between immigrants and other groups, such as US-born non-Hispanic Whites. Therefore, we exclude US-born persons from the sample.

A total of 171 910 individuals were interviewed by NHIS in 2011–2015. We limit the sample to households for whom the sample person is an immigrant born outside the territorial USA (excluding 141 174 observations), is a working-age adult (excluding 3856 observations), is not a pregnant woman (excluding 1648 observations), lives in a household that has income less than or equal to 300 % of the federal poverty line (excluding 8996 observations), and is not missing information on the food security module questions, health conditions or control variables (excluding 737 observations). The final sample consists of 15 499 immigrant adults at or below 300 % of the federal poverty line.

### Dependent variables

We examine five diet-related health outcomes: CHD, angina pectoris, heart attack, self-rated poor health and obesity. Clinical management and prevention of these diagnosed conditions include dietary recommendations^(^ ^22^ ^)^. While self-rated health is not technically classified as a heart-related health condition, we include it here since it is commonly used in the literature as an important health measure and highly correlated with the other heart-related health outcomes. Also, while the other measures are based on a diagnosis from a doctor, self-rated poor health allows respondents to give their subjective interpretation of how they feel and is not dependent on health-care access/utilization.

Self-rated health is measured by the individual’s response to the following question: ‘Would you say your health in general is excellent, very good, good, fair or poor?’ Self-rated poor health is a binary measure equal to 1 if the respondent reported his or her health was fair or poor, and 0 if the respondent reported his or her health was good, very good or excellent. Following National Institutes of Health clinical guidelines, we define obesity as BMI of 30 kg/m^2^ or above. Self-reported measures of height and weight are often misreported but maintaining a sample of non-elderly adults (18–64 years) and reporting results separately by gender should minimize the effects of reporting bias^(^ ^6^ ^)^. CHD, angina pectoris or heart attack are binary variables based on diagnosis by a medical professional.

### Food insecurity

The focal explanatory variable in the present study is household food insecurity. We obtain this variable from the ten-item US Adult Food Security Survey Module examining the household’s circumstances regarding food access over the past 30 d. Each question asks about the household’s circumstances or behaviours when having difficulty meeting their basic food needs due to a lack of economic resources. The sum of affirmed responses from the survey module provides the measure of the household’s food security status^(^ ^7^ ^,^ ^33^ ^)^. Households are then categorized into four groups: high food security, marginal food security, low food security and very low food security. For the analyses, we first use a binary measure of the household’s severity of food insecurity. Food insecurity is coded as 1 if a household experienced low or very low food security within the past 30 d; 0 otherwise. This measure captures experiences related to food insecurity that range in severity from reducing the quality and variety of food to experiencing physiological hunger. In subsequent analysis, we use a categorical version of food security status that includes the four groups: high food security, marginal food security, low food security and very low food security.

### Acculturation

The NHIS provides a categorical measure for length of US residence: less than 1 year, 1–4 years, 5–9 years, 10–14 years, and 15 years or more. To maintain appropriate sample size and variation in food security status (i.e. no immigrant in the ‘less than 1 year’ category reported being food insecure), we combined and recoded the first two categories as 0–4 years. Thus, the categorical measure of years in the USA includes the four groups: 0–4 years, 5–9 years, 10–14 years, and 15 years or more. Consistent with previous research (e.g. Antecol and Bedard and Commodore-Mensah *et al*.^(^ ^2^ ^,^ ^5^ ^)^), this information provides the primary measure of acculturation.

### Other control variables

Research shows gender plays an important role for both food insecurity and CHD and related health outcomes^(^ ^34^ ^)^. Women are particularly sensitive to food insecurity since they often act as household managers of food issues, sometimes depriving themselves in times of scarcity to feed other household members^(^ ^34^ ^–^ ^39^ ^)^. This behaviour can create health inequalities between women and men. Thus, in addition to the analysis using the total sample, we also decompose the sample by gender. The analysis includes additional controls for many demographic and socio-economic determinants of immigrant health: age, age-squared,* race and ethnicity, education level, marital status, size of family, number of children, any elderly persons present in household, the individual’s employment status, log household income,† citizenship status, health insurance coverage status and region of US residence. We use the individual’s ability to speak English well as a second marker of acculturation. English is an indicator variable equal to 1 if the individual speaks English well or very well.

The NHIS also collects information on the individual’s region of birth. This variable categorizes respondents’ region of birth in the following nine regions: Mexico, Central America, Caribbean Islands (MCC); South America (SA); Europe (EU); Russia and former Soviet Union areas (RSA); Africa (AF); the Middle East (ME); Indian subcontinent (IND); Central Asia (CA); and Southeast Asia (SEA). MCC is the largest immigrant group in the USA, making up roughly 68 % of the sample.

## Econometric methods

The overall goal is to answer the following two research questions. First, what is the relationship of household food insecurity and acculturation with CHD and related health outcomes? To answer this question, we estimate a series of multivariate logistic regression models examining the associations of household food insecurity and acculturation separately with each of the five health outcomes.‡ Second, how is food insecurity associated with these health outcomes over years of living in the USA? This step involves estimating the logistic regressions with an interaction term between food insecurity and acculturation. This reveals whether living longer in the USA moderates the relationship between food insecurity and poor heart-related health.

Consider the following model: (1)

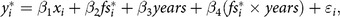
where 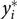 and 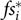 are latent binary variables representing the utility associated with an individual’s underlying health outcome and his or her household’s food security status, respectively. The *β*s represent the estimated regression coefficients; *x*_*i*_ contains exogenous variables to control for the common determinants of heart-related health outcomes *y*_*i*_, as described above; *years* represents the categorical measure of length of US residence (acculturation); 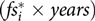 contains the interaction term of household food insecurity with the measure of length of USA residence; and 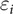 represents unobserved individual heterogeneity. Thus, the probability that an immigrant adult experiences CHD or a related health outcome is: (2)
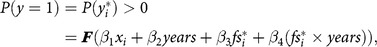
where ***F*** (·) is the logistic cumulative distribution function(3)
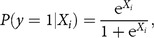

where 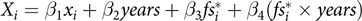 and 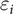 is assumed to be distributed with *var* (*ε*) = *π*^2^/3. We estimate these probabilities using maximum likelihood. Since the data are observational and cross-sectional, endogeneity may be of concern and causality cannot be inferred without strong assumptions. However, the findings reveal strong associations between the variables of interest.

## Results

**Fig. 1:**
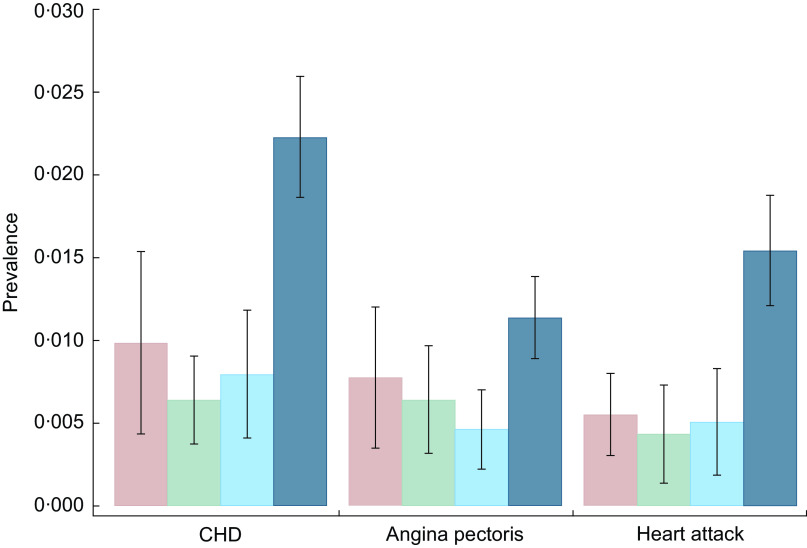
Prevalence of CHD, angina pectoris and heart attack over years of living in the USA (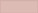, 0–4 years; 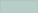, 5–9 years; 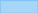, 10–14 years; 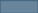, 15 years or more) among low-income, working-age immigrant adults. Data source is the pooled National Health Interview Survey 2011–2015. Values are proportions, with their se represented by vertical bars. Estimates account for complex survey design and confidence intervals are estimated at the 95 % level

**Fig. 2:**
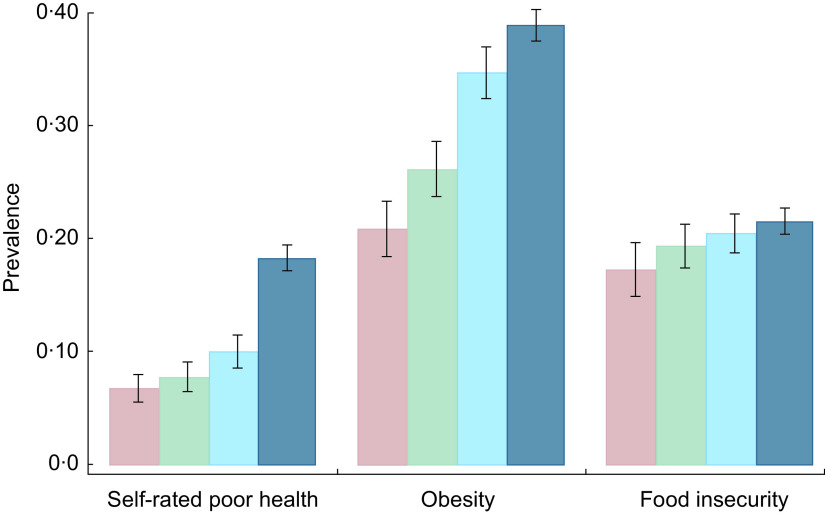
Prevalence of self-rated poor health, obesity and food insecurity over years of living in the USA (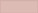, 0–4 years; 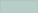, 5–9 years; 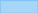, 10–14 years; 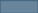, 15 years or more) among low-income, working-age immigrant adults. Data source is the pooled National Health Interview Survey 2011–2015. Values are proportions, with their se represented by vertical bars. Estimates account for complex survey design and confidence intervals are estimated at the 95 % level

Figures 1 and Fig. 2 show the prevalence of CHD, angina pectoris and heart attack (Fig. 1) and self-rated poor health, obesity and food insecurity (Fig. 2) over years of living in the USA. In the sample of low-income immigrant adults, 15 years or more in the USA is associated with a higher prevalence of all poor health and food insecurity indicators. Interestingly, for CHD, angina pectoris and heart attack, the prevalence rates are lower among those with 5–14 years in the USA than among those with 0–4 years in the USA.

Table 1 shows significant heterogeneity in the prevalence of food insecurity and CHD and related health outcomes by region of birth. The prevalence of food insecurity is highest for those born in MCC (24·6 %), followed by AF (21·9 %), EU (16·1 %) and SA (15·9 %). The prevalence of CHD is highest for immigrants from ME (3·2 %). Angina pectoris is highest among immigrants from EU (1·7 %), and heart attack is most prevalent among immigrants from ME (2·5 %). Self-rated poor health is highest for those born in MCC or ME (both 15·3 %). Lastly, the prevalence of obesity is highest for immigrants from MCC (41·2 %).

**Table 1:** Prevalence of CHD and related outcomes and food insecurity for low-income, working-age immigrant adults in the USA by region of birth

	Total sample	MCC	SA	EU	RSA	AF	ME	IND	CA	SEA
Food insecurity	0·206	0·246	0·159	0·161	0·098	0·219	0·099	0·088	0·058	0·133
se	0·005	0·006	0·015	0·017	0·031	0·022	0·028	0·013	0·010	0·011
CHD	0·016	0·016	0·013	0·028	0·003	0·003	0·032	0·021	0·011	0·011
se	0·001	0·001	0·004	0·007	0·003	0·002	0·011	0·007	0·004	0·004
Angina pectoris	0·009	0·009	0·005	0·017	0·006	0·001	0·010	0·011	0·006	0·007
se	0·001	0·001	0·003	0·007	0·005	0·001	0·005	0·005	0·00	0·00
Heart attack	0·011	0·011	0·007	0·015	0·006	0·008	0·025	0·014	0·010	0·007
se	0·001	0·001	0·003	0·005	0·006	0·005	0·010	0·005	0·005	0·003
Self-rated poor health	0·138	0·153	0·089	0·131	0·117	0·083	0·153	0·099	0·096	0·134
se	0·004	0·005	0·012	0·018	0·033	0·012	0·028	0·015	0·013	0·011
Obesity	0·342	0·412	0·243	0·335	0·223	0·313	0·282	0·152	0·103	0·142
se	0·005	0·006	0·015	0·022	0·054	0·022	0·035	0·017	0·013	0·014
Number of observations	15 499	9949	906	779	240	684	410	658	761	1112

Data source is the pooled National Health Interview Survey 2011–2015. Coefficients are proportions, with se given in the following row. All estimates account for complex survey design. Analysis sample includes households at or below 300 % of the federal poverty line. The immigrants’ region of birth are the following nine possible regions: Mexico, Central America, Caribbean Islands (MCC); South America (SA); Europe (EU); Russia and former Soviet Union areas (RSA); Africa (AF); the Middle East (ME); Indian subcontinent (IND); Central Asia (CA); and Southeast Asia (SEA).

Table 2 shows descriptive statistics for our sample of low-income immigrant adults and their households. The sample is evenly split between female and male respondents. The majority of the sample is Hispanic (65·1 %) and married or with a domestic partner (65·2 %). About 40 % of the sample has no high-school diploma. A minority of the sample are US citizens (38·7 %) and 30·4 % speak English well. About 8% of respondent households include a senior adult. A slight majority of respondents have health insurance (54·4 %). A majority of respondents have been in the USA for 15 years or more (54·6 %) and most are employed (67·7 %). The average age of respondents is 40 years, with about four members in the household and one child on average.

**Table 2:** Descriptive statistics of the sample population of low-income, working-age immigrant adults in the USA

Categorical explanatory variable	Frequency (*n*)	Proportion or se
Female	7807	0·504
se		0·005
Ethnicity		
Hispanic	10 097	0·651
se		0·008
Non-Hispanic White	1585	0·102
se		0·005
Non-Hispanic Black	1271	0·082
se		0·004
Non-Hispanic Asian or Other	2545	0·164
se		0·006
Marital status		
Married or domestic partner	10 110	0·652
se		0·005
Widowed, separated or divorced	1954	0·126
se		0·00
Never married	3434	0·222
se		0·005
Education status		
High-school dropout	6133	0·395
se		0·007
High-school graduate/GED	4000	0·258
se		0·005
More than high school	5366	0·347
se		0·007
US citizen	5996	0·387
se		0·007
Speaks English well	4707	0·304
se		0·005
At least one senior in household	1282	0·083
se		0·003
Health insurance coverage	8434	0·544
se		0·007
Years in the USA		
0–4 years	1718	0·111
se		0·004
5–9 years	2294	0·148
se		0·004
10–14 years	3022	0·195
se		0·004
15 years or more	8464	0·546
se		0·006
Person is employed	10 486	0·677
se		0·005
Continuous explanatory variable	Mean	
Age (years)	39·7	
se	0·158	
Size of family	3·8	
se	0·024	
Number of children	1·3	
se	0·017	
Log household income	10·7	
se	0·019	
Number of observations	15 499	

GED, General Educational Development.

Data source is the pooled National Health Interview Survey 2011–2015. Coefficients are proportions, with their se given in the following row. All estimates account for complex survey design. Analysis sample includes households at or below 300 % of the federal poverty line.

### Associations of food insecurity and acculturation with CHD and related outcomes

Next, we present OR from multivariate logistic regressions of household food insecurity and acculturation *v*. CHD and related health outcomes (without using the interaction term between food insecurity and acculturation). Estimation results for each of the five health outcomes appear in the top panels of Tables 3 and 4 among the full sample and in the sample divided by gender.* The interaction effects shown in the bottom panels are discussed in the next section. Overall, the results show a strong association between food insecurity and poor heart-related health, just as the stress from food insecurity and/or diet sensitivity of the conditions examined would suggest. Table 3 shows that food insecurity is associated with 57 % higher odds of being diagnosed with CHD, 81 % higher odds of angina pectoris and more than doubled odds of heart attack. For all three heart outcomes, the relationship with food insecurity is stronger for women and not significant for men. Number of years in the USA (acculturation) is statistically significant only for angina pectoris for the female sub-sample. The symbol † signifies where female coefficients are statistically significantly different from male coefficients. Gender differences in food insecurity are statistically significant for angina pectoris, heart attack and obesity; while gender differences in acculturation are statistically significant for angina pectoris, self-rated poor health and obesity. Female immigrants in the US for 5 years or more are less likely to have been diagnosed with angina pectoris than those in the US for less than 5 years (OR = 0·436, 0·202 and 0·325 for 5–9 years, 10–14 years and 15 years or more, respectively).

**Table 3:** Results from multivariate logistic regressions of household food insecurity and acculturation *v*. CHD, angina pectoris and heart attack among low-income, working-age immigrant adults in the USA

Variable	CHD			Angina pectoris				Heart attack		
	Full sample	Female	Male	Full sample	Female	Female – interaction model	Male	Full sample	Female	Male
Food insecurity	1·565***	1·857***	1·339	1·812***	2·502***,†	3·059	1·192	2·197***	4·046***,†	1·453
se	0·227	0·396	0·309	0·299	0·637	2·118	0·285	0·448	1·327	0·371
Years in the USA (Ref.: 0–4)										
5–9 years	0·885	1·030	0·652	0·992	0·436*,†	0·705	2·454	1·290	1·732	0·413
se	0·355	0·586	0·404	0·379	0·205	0·417	1·505	0·548	1·324	0·361
10–14 years	0·908	1·099	0·667	0·608	0·202***,†	0·349	2·107	1·290	1·053	1·228
se	0·383	0·562	0·399	0·245	0·112	0·227	1·267	0·579	0·883	0·868
15 years or more	1·130	1·100	1·032	0·706	0·325***,†	0·299***	1·787	1·778	1·193	1·969
se	0·404	0·613	0·505	0·229	0·120	0·135	0·972	0·708	0·981	1·188
Interaction										
Food insecurity × 5–9 years	–	–	–	–	–	0·269	–	–	–	–
se	–	–	–	–	–	0·246	–	–	–	–
Food insecurity × 10–14 years	–	–	–	–	–	0·100**	–	–	–	–
se	–	–	–	–	–	0·114	–	–	–	–
Food insecurity × 15 years or more	–	–	–	–	–	1·099	–	–	–	–
se	–	–	–	–	–	0·814	–	–	–	–
Interaction *F* statistic	–	–	–	–	–	3·58	–	–	–	–
Interaction *P* value	–	–	–	–	–	0·014	–	–	–	–
Number of observations	15 499	7807	7691	15 499	7807	7807	7691	15 499	7807	7691

Data source is the pooled National Health Interview Survey 2011–2015. Dependent variables are CHD, angina pectoris and heart attack, respectively. Coefficients are OR, with se given in the following row. All estimates account for complex survey design. Each regression also adjusts for age, age-squared, marital status, education level, size of family, number of children in the household, elderly person indicator, employment status, log household income, and year and region fixed effects. Reference categories (Ref.) are: in the USA for 0–4 years, White non-Hispanic, single/never married, more than a high-school education, 2011 and Northwest. Analysis sample includes households at or below 300 % of the federal poverty line.

* Significant at the 10 % level.

** Significant at the 5 % level.

*** Significant at the 1 % level.

† Female coefficient statistically different from the male coefficient.

**Table 4:** Results from multivariate logistic regressions of household food insecurity and acculturation *v*. self-rated poor health and obesity among low-income, working-age immigrant adults in the USA

Variable	Self-rated poor health				Obesity			
	Full sample	Full sample – interaction model	Female	Male	Full sample	Full sample – interaction model	Female	Male
Food insecurity	2·801***	4·651***	2·931***	2·704***	1·260***	1·785***	1·336***,†	1·204**
se	0·180	1·127	0·249	0·252	0·065	0·346	0·096	0·088
Years in the USA (Ref.: 0–4)								
5–9 years	1·253*	1·387*	1·038	1·517*	1·117	1·226*	1·041	1·188
se	0·164	0·255	0·183	0·359	0·115	0·137	0·144	0·182
10–14 years	1·497***	1·867***	1·219	1·818***	1·422***	1·600***	1·166†	1·706***
se	0·212	0·358	0·229	0·419	0·132	0·160	0·146	0·241
15 years or more	1·761***	2·130***	1·363*,†	2·320***	1·478***	1·591***	1·453***	1·475***
se	0·234	0·376	0·231	0·554	0·137	0·166	0·186	0·200
Interaction								
Food insecurity × 5–9 years	–	0·734	–	–	–	0·664*	–	–
se	–	0·228	–	–	–	0·151	–	–
Food insecurity × 10–14 years	–	0·529**	–	–	–	0·596**	–	–
se	–	0·156	–	–	–	0·125	–	–
Food insecurity × 15 years or more	–	0·575**	–	–	–	0·722	–	–
se	–	0·143	–	–	–	0·147	–	–
Interaction *F* statistic	–	2·11	–	–	–	2·31	–	–
Interaction *P* value	–	0·099	–	–	–	0·077	–	–
Number of observations	15 499	15 499	7807	7691	15 499	15 499	7807	7691

Data source is the pooled National Health Interview Survey 2011–2015. Dependent variables are self-rated poor health and obesity, respectively. Coefficients are OR, with se given in the following row. All estimates account for complex survey design. Each regression also adjusts for age, age-squared, marital status, education level, size of family, number of children in the household, elderly person indicator, employment status, log household income, and year and region fixed effects. Reference categories are: in the USA for 0–4 years, White non-Hispanic, single/never married, more than a high-school education, 2011 and Northwest. Analysis sample includes households at or below 300 % of the federal poverty line.

* Significant at the 10 % level.

** Significant at the 5 % level.

*** Significant at the 1 % level.

† Female coefficient statistically significantly different from male coefficient.

Table 4 shows that food insecurity is associated with a much higher likelihood of reporting poor health for both women and men (OR = 2·931 and 2·704, respectively). Food insecurity is also associated with a higher likelihood of being obese for both women and men (OR = 1·336 and 1·204, respectively) with an OR of 1·260 for the full sample. Length of time in the USA is significantly associated with both self-rated poor health and obesity. Among women, only those in the USA for 15 years or more are significantly more likely to report poor health than those in the USA less than 5 years (OR = 1·363). Among men, respondents in the USA for 5 or more years are more likely to report poor health than those in the USA for less than 5 years, with a higher likelihood of poor health for each increasing time category (OR = 1·517, 1·818 and 2·320 for 5–9 years, 10–14 years and 15 years or more, respectively). For obesity, female immigrants in the USA for 15 years or more are more likely to be obese than those in the USA less than 5 years (OR = 1·453). Male immigrants in the USA for 10 or more years are more likely to be obese than immigrants in the USA for less than 5 years (OR = 1·706 and 1·475 for 10–14 years and 15 years or more, respectively).

### Association of food insecurity with CHD and related outcomes over years in the USA

**Fig. 3:**
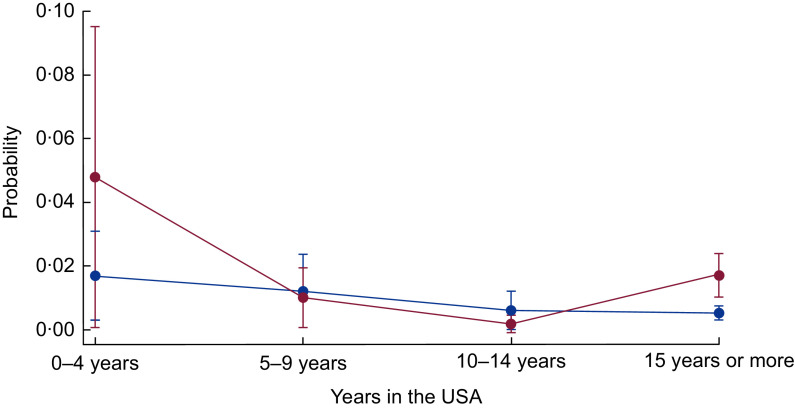
Probability of angina pectoris over years of living in the USA by food security status (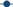, food secure; 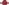, food insecure) among low-income, working-age immigrant women. Data source is the pooled National Health Interview Survey 2011–2015. Values are probabilities, with their se represented by vertical bars. Estimates account for complex survey design and confidence intervals are estimated at the 95 % level

**Fig. 4:**
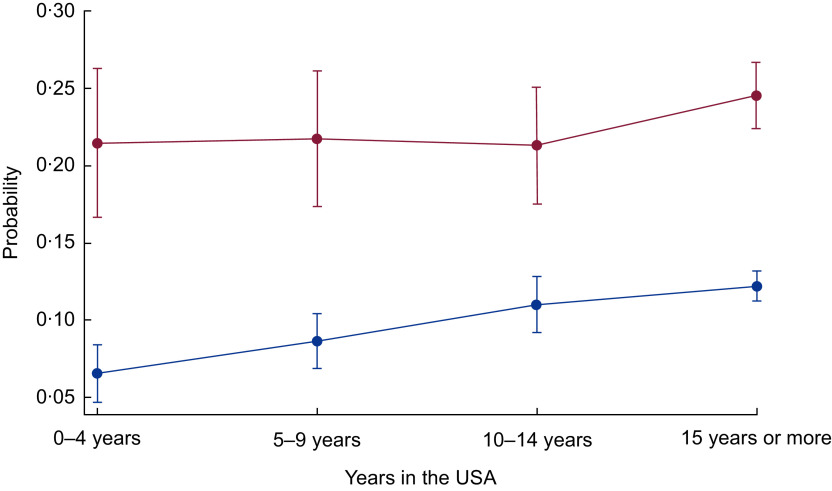
Probability of self-rated poor health over years of living in the USA by food security status (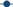, food secure; 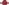, food insecure) among low-income, working-age immigrant adults. Data source is the pooled National Health Interview Survey 2011–2015. Values are probabilities, with their se represented by vertical bars. Estimates account for complex survey design and confidence intervals are estimated at the 95 % level

**Fig. 5:**
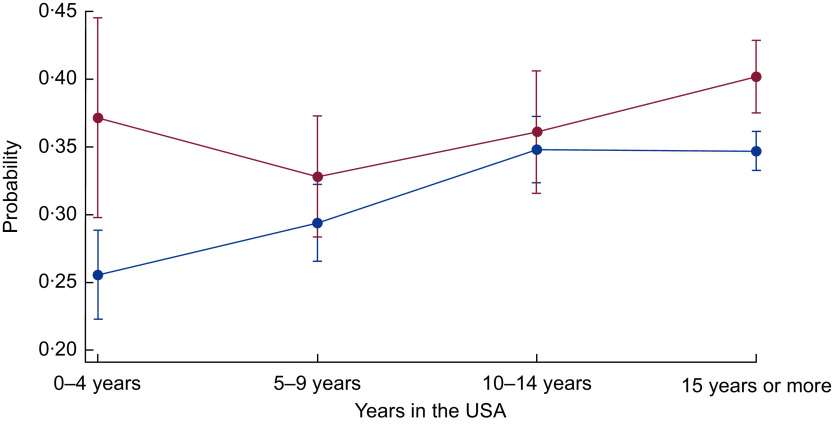
Probability of obesity over years of living in the USA by food security status (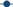, food secure; 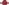, food insecure) among low-income, working-age immigrant adults. Data source is the pooled National Health Interview Survey 2011–2015. Values are probabilities, with their se represented by vertical bars. Estimates account for complex survey design and confidence intervals are estimated at the 95 % level

Next, in the bottom panels of Tables 3 and 4, we present the interactive associations of food insecurity and years in the USA on the health of immigrants. Specifically, since the dependent variable is measured in the odds metric (multiplicative effect), the interaction term is considered to be a ratio of OR. We present the results of the interaction models for those outcomes where food insecurity or acculturation were statistically significant without interaction terms (Tables 3 and 4). Table 3 shows that the interaction of food insecurity and acculturation is statistically significant for angina pectoris among female immigrants (interaction *P* value = 0·014). When the interaction is included, food insecurity alone is no longer statistically significant, nor are the OR for 5–9 years or 10–14 years in the USA. Those who are food insecure and in the county for 10–14 years are less likely to be diagnosed with angina pectoris (OR = 0·100). As depicted in Fig. 3, for female immigrants in the USA for less than 5 years or more than 15 years, food insecurity is related to a higher probability of angina pectoris, but not for those in the USA for 5–14 years. Table 4 shows the interaction between food insecurity and time in the USA is statistically significant for poor health and obesity for the full sample (interaction *P* value = 0·099 and 0·077, respectively). The interaction for poor health suggests that being food insecure somewhat reduces the higher likelihood of poor health for those in the USA for more than 10 years (OR = 0·529–0·575). As shown in Fig. 4, the difference in the probability of poor health by food security status is wider for newly arrived immigrants, narrows for those who have lived in the USA for 5–14 years, but is larger again for those in the USA for 15 or more years. Similarly, for obesity, food insecurity has an attenuating effect on the higher likelihood of obesity among those in the USA from 5 to 14 years (OR = 0·596–0·664). Figure 5 shows the gap in the probability of obesity by food security status is greatest for those in the USA less than 5 years, and for those in the USA for 15 years or more. Generally, Figs 3 to 5 show that the difference in probability of poor health, obesity and women’s angina pectoris by food security status narrows from 5 to 14 years, but is widest for those in the USA less than 5 years and those in the USA more than 15 years. Thus, more recent and long-term food-insecure immigrants are more vulnerable to CHD and related health outcomes than those in the USA for 5–14 years.

### Heterogeneity across the severity of food insecurity

In this section we ask a follow-up question: Does the relationship between household food insecurity and CHD and related health outcomes change with the severity of food insecurity? It is possible that the most severe cases drive the association between food insecurity and poor heart-related health. Table 5 shows the OR using the categorical version of food security status which includes the four groups: high food security, marginal food security, low food security and very low food security. For each logistic model, we compare high food security with the results in each other category.

**Table 5:** Results from multivariate logistic regressions of categorical food security status *v*. CHD and related health outcomes among low-income, working-age immigrant adults in the USA

	CHD	Angina pectoris	Heart attack	Self-rated poor health	Obesity
Food security status (Ref.: high food security)					
Marginal food security	1·505*	2·172***	0·698†,‡	1·878†,‡	1·227***
se	0·342	0·467	0·206	0·151	0·080
Low food security	1·827***	2·035***	2·213***	2·638***,§	1·339***
se	0·337	0·434	0·550	0·199	0·086
Very low food security	1·497*	2·549***	1·795**	4·786***	1·267***
se	0·330	0·649	0·490	0·452	0·100
Years in the USA (Ref.: 0–4)					
5–9 years	0·883	0·993	1·286	1·265*	1·117
se	0·354	0·380	0·551	0·166	0·115
10–14 years	0·904	0·610	1·286	1·509***	1·425***
se	0·380	0·247	0·579	0·216	0·133
15 years or more	1·130	0·710	1·787	1·776***	1·486***
se	0·402	0·228	0·723	0·236	0·138
Number of observations	15 472	7793	7679	15 472	7793

Data source is the pooled National Health Interview Survey 2011–2015. Dependent variables are CHD, angina pectoris, heart attack, self-rated poor health and obesity, respectively. Coefficients are OR, with se given in the following row. All estimates account for complex survey design. Each regression also adjusts for age, age-squared, marital status, education level, size of family, number of children in the household, elderly person indicator, employment status, log household income, and year and region fixed effects. Reference categories (Ref.) are: in the USA for 0–4 years, White non-Hispanic, single/never married, more than a high-school education, 2011 and Northwest. Analysis sample includes households at or below 300 % of the federal poverty line.

* Significant at the 10 % level.

** Significant at the 5 % level.

*** Significant at the 1 % level.

† Marginal food security is statistically significantly different from the low food security category.

‡ Marginal food security is statistically significantly different from the very low food security category.

§ Low food security is statistically significantly different from the very low food security category.

Overall, the results in Table 5 show that each category of food insecurity is strongly associated with CHD and related health outcomes among the study sample. However, the probability of CHD and related outcomes do not increase as the severity of food insecurity worsens – except for poor health. The highest likelihood of CHD, heart attack and obesity is among those with low food security (OR = 1·339–2·213). Low food security primarily signals a reduction in dietary quality or variety. The highest likelihood of angina pectoris is for those with very low food security. Very low food security means not only a reduction in dietary quality, but also a reduction in dietary quantity. The increasing severity of food insecurity is more linearly related to self-rated poor health: OR = 1·878 for marginal food security, OR = 2·638 for low food security and OR = 4·786 for very low food security.

Table 5 also shows whether the differences between marginal, low and very low food security are statistically significant (†, ‡ and §). The symbol † shows comparison tests between respondents in marginal and low food secure households; the symbol ‡ shows comparison tests between respondents in marginal and very low food secure households; and the symbol § shows comparison tests between respondents in low and very low food secure households. Differences are statistically significant only for the heart attack and poor health models. For the odds of a heart attack, marginal food security is statistically different from low and very low food security. The odds of poor health are statistically different across each of the food security categories.

### Robustness and sensitivity analysis

We also check the robustness and sensitivity of the results (see appendices). First, we examine whether BMI mediates the results discussed above and include it as a covariate in the models for all outcomes, except obesity (Appendix 1 and Appendix 2). The coefficient for BMI is statistically significant and positive (OR greater than 1) in models for CHD, heart attack and poor health. However, the results for food insecurity and years in the USA are substantively unchanged from the models that do not include BMI (see Tables 3 and 4). There is no evidence that the relationship between food insecurity and health outcomes, or time in USA and health outcomes, operates through obesity.

Second, since immigrants from the MCC region represent a large majority of the sample (68 %), we test whether the results change if these individuals were dropped from the sample (Appendix 3 and Appendix 4). The biggest effect of dropping the MCC respondents from the sample is that fewer coefficients are significant, most likely due to the loss in sample size and statistical power. In general, the directions of the coefficients and magnitude are the same whether the whole sample is used or only non-MCC immigrants. The only substantive change in results is for the male model for angina pectoris outcome. The coefficient for food insecurity changes direction and is statistically significant in the non-MCC immigrant male model, indicating that food insecurity is associated with a lower likelihood of being diagnosed with angina pectoris for non-MCC immigrant men.

In all robustness and sensitivity checks, we find that generally the relationships between food insecurity, acculturation, and CHD and related outcomes do not change appreciably. The magnitude and direction of the food insecurity and acculturation coefficients remain relatively the same. Thus, the results are robust across chosen variables and samples.

## Discussion

Using repeated cross-sectional data from the NHIS from 2011–2015, we address two research questions. First, what is the relationship of household food insecurity and acculturation with diagnosis of CHD and related health outcomes, namely angina pectoris, heart attack, self-rated poor health and obesity? Second, what is the association of food insecurity with these health outcomes over years of living in the USA? Overall, we find that both food insecurity and acculturation are strongly associated with CHD and related health outcomes among working-age immigrant adults (research question 1). This is not surprising since diet sensitivity is a common factor among these conditions and food insecurity increases the difficulty of following a healthy diet, given limitations on money to afford healthy foods and time to cook healthy meals^(^ ^22^ ^,^ ^29^ ^,^ ^44^ ^)^. This also corresponds to previous research linking length of US residence with negative health outcomes among immigrant adults^(^ ^10^ ^,^ ^45^ ^,^ ^46^ ^)^ and suggests that dietary acculturation may increase over time in the USA. For example, previous research consistently shows a strong association between acculturation and unhealthy food intake, such as higher saturated fat, sugar and cholesterol^(^ ^10^ ^,^ ^47^ ^–^ ^49^ ^)^.

We also find significant gender heterogeneity in these relationships, where food insecurity is associated with CHD and related outcomes of female immigrants more than male. One explanation for these findings is that the determinants of heart-related health outcomes are gender specific^(^ ^34^ ^,^ ^50^ ^,^ ^51^ ^)^. Women may also be more susceptible to negative impacts of food insecurity. For example, Martin and Lippert^(^ ^38^ ^)^ find that mothers are at greater risk for obesity than child-free women, or men regardless of parenthood status, because mothers often prioritize their children’s access to healthy food over their own.

Lastly, we find a significant interactive association between food insecurity and years in the USA for self-rated health, obesity and women’s angina pectoris. Generally, the significant interactions suggest that food insecurity is associated more strongly with CHD and related outcomes for immigrants in the USA for less than 5 years or those in the USA for 15 years or more, than those in the USA for 5–14 years. This is an important finding and provides further detail on the relationship between food insecurity, acculturation and health than previous literature. For example, Ryan-Ibarra *et al*.^(^ ^51^ ^)^ found among Californian immigrant women that food insecurity was associated with a higher prevalence of obesity for those in the USA for 10 years or longer, but was statistically insignificant for those in the USA for less. Using a nationally representative sample and our more granular categorical version of years in the USA provides more information about those in the USA from 5 to 14 years, than using a simple binary measure. It is worth noting that in earlier analyses when we used a dichotomous measure of time in the USA (less than 15 years; 15 years or more) our findings were similar to those of Ryan-Ibarra and colleagues. We found it was important to model time in the USA with more categories because there were important differences over time that were masked otherwise. Future research using a continuous measure of time in the USA may provide even further insights into these relationships.

There are several possible reasons why food insecurity has a greater association with poor health for those in the USA for under 5 years. First, having recently migrated, immigrants would most likely be under more stress than those who immigrated 5 or more years ago; stress from the move and stress from adapting to a new environment^(^ ^52^ ^)^. For example, linguistic isolation (inability to speak English well) and abrupt changes to nutritional sources have been shown to increase obesity and poor health among immigrants^(^ ^53^ ^–^ ^55^ ^)^. However, living in immigrant enclaves with Spanish-speaking neighbours and options to acquire traditional foods is associated with healthy dietary patterns among Hispanic immigrants^(^ ^53^ ^)^. Hence, it may take some time for recent immigrants to find their enclave. Another likely reason why food insecurity plays a bigger role in determining health for immigrants in the USA for less than 5 years is their ineligibility for federal assistance programmes. Legal immigrants must wait 5 years before being eligible for federal programme benefits, unless they have children or are disabled^(^ ^56^ ^,^ ^57^ ^)^. This means that immigrants in the USA for less than 5 years may be unable to participate in food assistance programmes such as the Supplemental Nutrition Assistance Program (SNAP) and the Special Supplemental Nutrition Program for Women, Infants, and Children.

For immigrants in the USA for 15 years or more, we find consistent evidence with the existing literature, in that acculturation is associated with poorer health outcomes and that food insecurity may be an important pathway through which this occurs^(^ ^55^ ^)^. While immigration might initially take place with the goal of increasing welfare over time, in fact years of residence in the USA correlate with declining health outcomes among immigrant adults from various backgrounds and countries of origin^(^ ^58^ ^–^ ^60^ ^)^. For example, Koya and Egede^(^ ^61^ ^)^ found that acculturation predicts increased rates of tobacco use, hyperlipidaemia and obesity. Similarly, Oza-Frank and Narayan^(^ ^60^ ^)^ found that length of time in the USA correlates with higher diabetes prevalence, independent of age and obesity. Adoption of health-related behaviours, such as unhealthy diets and sedentary lifestyles of the dominant culture, can strongly affect immigrant health over time^(^ ^6^ ^,^ ^45^ ^,^ ^62^ ^–^ ^65^ ^)^. Immigrants’ dietary acculturation in the USA typically involves increases in consumption of energy-dense, processed foods and animal products, and decreases in consumption of fruits, vegetables and whole grains^(^ ^46^ ^,^ ^66^ ^,^ ^67^ ^)^. Research on the level of dietary acculturation also shows a link between increased acculturation and chronic disease risk factors^(^ ^47^ ^,^ ^48^ ^,^ ^66^ ^–^ ^70^ ^)^.

It is possible that immigrants in the USA for 5–14 years have relatively less stress and have figured out how to navigate US systems and found friends and neighbours they can rely on in times of need but have not fully acculturated to poorer diets. Further research is needed using different data and alternative health measures to understand more fully how years in the USA interact with food insecurity.

### Limitations

The results of the present study should be interpreted in the context of several limitations. First, measures of CHD and related health outcomes in the NHIS are self-reported and subject to reporting bias. However, the survey is nationally representative, has been extensively validated and is the key data source for information on the nation’s health. Second, the pooled cross-sectional nature of the data limits the analyses. Cross-sectional data do not allow for the examination of longitudinal change of health over time and may conflate cohort effects with the effects of time in the USA. Longitudinal data would allow the analysis to address the effects of acculturation more directly, by taking into account the life experiences of immigrants before migration and by controlling for socio-economic, demographic and cultural confounders; similar limitations have characterized other acculturation-related research^(^ ^48^ ^,^ ^69^ ^)^. For example, since we are limited by the categories of years of US residence (0–4 years, 5–9 years, 10–14 years, 15 years or more), we must treat each category as if health characteristics remain constant within. However, some immigrants may arrive with greater exposure to American lifestyle behaviours than others and selection processes differ in important ways^(^ ^71^ ^–^ ^75^ ^)^. Third, due to data limitations we are unable to examine the direct mechanisms of the impact of food insecurity on immigrant health. It is unclear whether food insecurity is related to CHD and related health outcomes due to dietary impacts, stress or other factors. Also noteworthy is that the food security module in the NHIS has only a 30 d recall period. Longitudinal data with multiple observations of food insecurity and health may show a more complex relationship among health, food insecurity and time in the USA. However, the short recall period for food insecurity offers advantages in terms of reliability and still reveals strong associations with contemporaneous food insecurity and dynamic health outcomes.

### Policy implications

The present study is relevant to contemporary questions about how public health policy might affect health outcomes in the USA. First, the associations between food insecurity and CHD and related health outcomes suggest reducing food insecurity may save health-care costs. Second, the results are relevant to a recent call for clinical screenings of food insecurity^(^ ^76^ ^)^. Knowing the food security status of immigrant patients who are suffering from or predisposed to CHD and related outcomes may be important to helping them manage these conditions^(^ ^22^ ^)^. Third, recent research has shown that SNAP participation significantly reduces the likelihood of reporting poor health in low-income households^(^ ^77^ ^,^ ^78^ ^)^. SNAP is one vehicle to reducing food insecurity, but there are rules that prevent some immigrants from receiving SNAP^(^ ^79^ ^)^. Future research could examine the possible effects of SNAP on the heart-related health of immigrants over years of US residence. Understanding the relationships between food insecurity, acculturation, and CHD and related health outcomes among immigrants can inform the development of public health and nutrition assistance programmes for the entire US population.

## Footnotes

† See Antecol and Bedard^(^ ^2^ ^)^ for an excellent review.

‡ Acculturation is formally defined as the process of cultural and psychological adjustment that occurs because of contact between two or more cultures^(^ ^3^ ^,^ ^4^ ^)^. Researchers (e.g. Commodore-Mensah *et al*.^(^ ^5^ ^)^) have often used length of residence in the USA as a proxy for acculturation, as we do here.

* We chose a polynomial age specification after also testing linear and spline specifications.

† Household income *x** was transformed using the inverse hyperbolic sine transformation,

‡ We also estimated binary choice with endogenous treatment models but found similar results. Additionally, these two-stage models showed that the correlation coefficient (*ρ*) of the errors between the two equations was consistently statistically insignificant. This validates the use of a single-equation non-linear probability model since it is more statistically efficient (i.e. it involves only one set of se). As a robustness check, we followed this analysis with a non-parametric bounds approach^(^ ^40^ ^–^ ^43^ ^)^. The non-parametric bounds estimates provided empirical support for the robustness of the logistic regressions – providing similar results – and for the validity of self-reported food security status. Results are available from the authors upon request.

* After testing the estimated coefficients, we found evidence that the covariates varied significantly by gender, justifying the gender-separated models.

## Appendix 1 *Robustness and sensitivity analysis: results from multivariate logistic regressions, controlling for BMI, of household food insecurity and acculturation* v. *CHD, angina pectoris and heart attack among low-income, working-age immigrant adults in the USA*

**Table:**

Variable	CHD			Angina pectoris			Heart attack		
	Full sample	Female	Male	Full sample	Female	Male	Full sample	Female	Male
Food insecurity	1·558***	1·841***	1·354	1·809***	2·485***	1·195	2·193***	3·837***	1·453
se	0·225	0·394	0·314	0·298	0·634	0·285	0·442	1·216	0·370
Years in the USA (Ref.: 0–4)									
5–9 years	0·874	1·013	0·657	0·985	0·430*	2·441	1·280	1·636	0·413
se	0·353	0·578	0·409	0·377	0·201	1·494	0·549	1·283	0·361
10–14 years	0·903	1·085	0·675	0·608	0·200***	2·103	1·305	1·030	1·229
se	0·382	0·558	0·404	0·246	0·110	1·262	0·587	0·902	0·870
15 years or more	1·134	1·095	1·047	0·708	0·324***	1·782	1·821	1·225	1·970
se	0·406	0·613	0·514	0·230	0·120	0·967	0·743	1·040	1·189
BMI	1·007**	1·005	1·011*	1·004	1·004	1·004	1·012*	1·016**	1·000
se	0·003	0·004	0·006	0·003	0·005	0·006	0·007	0·008	0·006
Number of observations	15 499	7807	7691	15 499	7807	7691	15 499	7807	7691

Data source is the pooled National Health Interview Survey 2011–2015. Dependent variables are CHD, angina pectoris and heart attack, respectively. Coefficients are OR, with se given in the following row. All estimates account for complex survey design. Each regression also adjusts for age, age-squared, marital status, education level, size of family, number of children in the household, elderly person indicator, employment status, log household income, and year and region fixed effects. Reference categories (Ref.) are: in the USA for 0–4 years, White non-Hispanic, single/never married, more than a high-school education, 2011 and Northwest. Analysis sample includes households at or below 300 % of the federal poverty line.

* Significant at the 10 % level.

** Significant at the 5 % level.

*** Significant at the 1 % level.

## Appendix 2 *Robustness and sensitivity analysis: OR from multivariate logistic regressions, controlling for BMI, of household food insecurity and acculturation* v. *self-rated poor health among low-income, working-age immigrant adults in the USA*

**Table:**

Variable	Self-rated poor health		
	Full sample	Female	Male
Food insecurity	2·800***	2·914***	2·727***
se	0·182	0·252	0·256
Years in the USA (Ref.: 0–4)			
5–9 years	1·251*	1·046	1·495*
se	0·166	0·189	0·353
10–14 years	1·499***	1·224	1·824**
se	0·214	0·232	0·424
15 years or more	1·778***	1·380*	2·346***
se	0·239	0·239	0·570
BMI	1·010***	1·009***	1·011***
se	0·002	0·002	0·003
Number of observations	15 499	7807	7691

Data source is the pooled National Health Interview Survey 2011–2015. Dependent variable is self-rated poor health. Coefficients are OR, with se given in the following row. All estimates account for complex survey design. Each regression also adjusts for age, age-squared, marital status, education level, size of family, number of children in the household, elderly person indicator, employment status, log household income, and year and region fixed effects. Reference categories (Ref.) are: in the USA for 0–4 years, White non-Hispanic, single/never married, more than a high-school education, 2011 and Northwest. Analysis sample includes households at or below 300 % of the federal poverty line.

* Significant at the 10 % level.

** Significant at the 5 % level.

*** Significant at the 1 % level.

## Appendix 3 *Robustness and sensitivity analysis: results from multivariate logistic regressions, dropping Mexican immigrants from the sample, of household food insecurity and acculturation* v. *CHD, angina pectoris and heart attack among low-income, working-age immigrant adults in the USA*

**Table:**

Variable	CHD			Angina pectoris			Heart attack		
	Full sample	Female	Male	Full sample	Female	Male	Full sample	Female	Male
Food insecurity	1·476	1·784	1·404	1·102	3·071**	0·314**	2·101**	5·985***	1·020
se	0·434	0·751	0·585	0·411	1·692	0·167	0·759	3·404	0·518)
Years in the USA (Ref.: 0–4)									
5–9 years	1·218	1·105	0·896	0·630	0·175*	1·090	0·882	0·636	0·123
se	0·807	1·361	0·856	0·455	0·159	1·133	0·736	0·792	0·162
10–14 years	1·186	1·134	1·013	0·781	0·206	1·314	1·392	0·886	0·537
se	0·914	1·552	0·781	0·548	0·225	1·155	1·057	1·199	0·642
15 years or more	2·619	3·599	1·598	0·607	0·358	0·537	2·769*	0·745	2·462
se	1·627	4·510	1·162	0·408	0·325	0·443	1·675	0·930	2·256
Number of observations	5844	2868	2681	5844	2868	2681	5844	2868	2681

Data source is the pooled National Health Interview Survey 2011–2015. Dependent variables are CHD, angina pectoris and heart attack, respectively. Coefficients are OR, with se given in the following row. All estimates account for complex survey design. Each regression also adjusts for age, age-squared, marital status, education level, size of family, number of children in the household, elderly person indicator, employment status, log household income, and year and region fixed effects. Reference categories (Ref.) are: in the USA for 0–4 years, White non-Hispanic, single/never married, more than a high-school education, 2011 and Northwest. Analysis sample includes households at or below 300 % of the federal poverty line and excludes individuals born in Mexico.

* Significant at the 10 % level.

** Significant at the 5 % level.

*** Significant at the 1 % level.

## Appendix 4 *Robustness and sensitivity analysis: results from multivariate logistic regressions, dropping Mexican immigrants from the sample*, *of household food insecurity and acculturation* v. *self-rated poor health and obesity among low-income, working-age immigrant adults in the USA*

**Table:**

Variable	Self-rated poor health			Obesity		
	Full sample	Female	Male	Full sample	Female	Male
Food insecurity	3·728***	4·320***	3·236***	1·442***	1·603***	1·301
se	0·499	0·763	0·633	0·154	0·220	0·226
Years in the USA (Ref.: 0–4)						
5–9 years	1·223	0·879	1·855	1·083	1·019	1·175
se	0·297	0·257	0·820	0·181	0·240	0·292
10–14 years	2·357***	1·746*	3·308***	1·403**	1·161	1·712**
se	0·593	0·542	1·334	0·225	0·265	0·408
15 years or more	3·169***	2·191***	5·018***	1·736***	1·514*	2·075***
se	0·707	0·644	1·817	0·279	0·358	0·478
Number of observations	5844	2868	2681	5844	2868	2681

Data source is the pooled National Health Interview Survey 2011–2015. Dependent variables are self-rated health and obesity, respectively. Coefficients are OR, with se given in the following row. All estimates account for complex survey design. Each regression also adjusts for age, age-squared, marital status, education level, size of family, number of children in the household, elderly person indicator, employment status, log household income, and year and region fixed effects. Reference categories (Ref.) are: in the USA for 0–4 years, White non-Hispanic, single/never married, more than a high-school education, 2011 and Northwest. Analysis sample includes households at or below 300 % of the federal poverty line and excludes individuals born in Mexico.

* Significant at the 10 % level.

** Significant at the 5 % level.

*** Significant at the 1 % level.
